# Role of Uropathogenic *Escherichia coli* Virulence Factors in Development of Urinary Tract Infection and Kidney Damage

**DOI:** 10.1155/2012/681473

**Published:** 2012-03-08

**Authors:** Justyna Bien, Olga Sokolova, Przemyslaw Bozko

**Affiliations:** ^1^Witold Stefanski Institute of Parasitology of the Polish Academy of Sciences, 51/55 Twarda Street, 00818 Warsaw, Poland; ^2^Institute of Experimental Internal Medicine, Otto von Guericke University, Leipziger Straße 44, 39120 Magdeburg, Germany; ^3^Department of Internal Medicine I, Faculty of Medicine, University of Tübingen, Otfried-Müller-Straße 10, 72076 Tübingen, Germany

## Abstract

Uropathogenic *Escherichia coli* (UPEC) is a causative agent in the vast majority of urinary tract infections (UTIs), including cystitis and pyelonephritis, and infectious complications, which may result in acute renal failure in healthy individuals as well as in renal transplant patients. UPEC expresses a multitude of virulence factors to break the inertia of the mucosal barrier. In response to the breach by UPEC into the normally sterile urinary tract, host inflammatory responses are triggered leading to cytokine production, neutrophil influx, and the exfoliation of infected bladder epithelial cells. Several signaling pathways activated during UPEC infection, including the pathways known to activate the innate immune response, interact with calcium-dependent signaling pathways. Some UPEC isolates, however, might possess strategies to delay or suppress the activation of components of the innate host response in the urinary tract. Studies published in the recent past provide new information regarding how virulence factors of uropathogenic *E. coli* are involved in activation of the innate host response. Despite numerous host defense mechanisms, UPEC can persist within the urinary tract and may serve as a reservoir for recurrent infections and serious complications. Presentation of the molecular details of these events is essential for development of successful strategies for prevention of human UTIs and urological complications associated with UTIs.

## 1. Introduction

*Escherichia coli* is a common inhabitant of the gastrointestinal tract of humans and animals. Usually, *E. coli* forms a beneficial symbiotic relationship with its host and plays important roles in promoting the stability of the luminal microbial flora and in maintaining normal intestinal homeostasis [1]. As a commensal, *E. coli* rather remains harmlessly confined to the intestinal lumen and rarely causes a disease. However, in the debilitated or immunosuppressed host, or when the gastrointestinal barriers are violated, even nonpathogenic-commensal strains of *E. coli *can cause infection [2]. Some strains of *E. coli *can diverge from their commensal cohorts, taking on a more pathogenic nature. These strains acquire specific virulence factors (via DNA horizontal transfer of transposons, plasmids, bacteriophages, and pathogenicity islands), which confer an increased ability to adapt to new niches and allow the bacteria to increase the ability to cause a broad spectrum of diseases.

The pathogenic *E. coli* strains are broadly classified as either enteric/diarrheagenic *E. coli* or extraintestinal *E. coli *(ExPEC). Six different *E. coli *“pathotypes,” including enteropathogenic *E. coli* (EPEC), enterohemorrhagic *E. coli* (EHEC), enterotoxigenic *E. coli* (ETEC), enteroaggregative *E. coli* (EAEC), enteroinvasive *E. coli* (EIEC), and diffusely adherent *E. coli* (DAEC), are the enteric/diarrheagenic *E. coli*, and two pathotypes, neonatal meningitis *E. coli *(NMEC) and uropathogenic *E. coli *(UPEC), are the most common ExPEC [2, 3]. Several pathotypes of enteric/diarrheagenic *E. coli* give rise to gastroenteritis but rarely cause disease outside of the intestinal tract. On the other hand, the ExPEC strains maintain the ability to exist in the gut without consequence but have the capacity to disseminate and colonize other host niches including the blood, the central nervous system, and the urinary tract, resulting in disease [4].

Urinary tract infections (UTIs) are considered to be the most common infections in humans. The development of UTIs depends on anatomical factors, the integrity of host defense mechanisms, and the virulence of the infecting organisms [5]. UTIs are classified into disease categories according to the site of infection: cystitis (the bladder), pyelonephritis (the kidney) and bacteriuria (the urine) [6]. Successful establishment of infection by bacterial pathogens requires adhesion to host cells, colonization of tissues, and, in certain cases, cellular invasion, followed by intracellular multiplication, dissemination to other tissues, or persistence. Colonization of the urine in the absence of the clinical symptoms is called asymptomatic bacteriuria (ABU). Most patients with ABU do not need treatment, and in many cases the colonizing by the ABU strains may help to prevent infection by other more virulent bacteria [7–9]. The primary causative agents responsible for more than 80% of all UTIs, including both ABU and symptomatic UTIs, are strains of uropathogenic *E. coli* [10–12].

UPEC is the cause of community-acquired UTIs and a large portion of nosocomial UTIs, accounting for substantial medical costs and morbidity and mortality worldwide [6, 13–15]. The ability of UPEC to cause symptomatic UTIs is associated with expression of a broad spectrum of virulence factors [16], with adhesive molecules being arguably the most important determinants of pathogenicity [17].

In contrast to symptomatic UPEC, the reason why ABU patients do not develop symptoms is not properly understood. However, it has been explained by a number of observations that many ABU strains are nonadherent and nonhaemolytic [18–20]. The strain *E. coli* 83972, which was isolated from a patient with ABU who had carried it for 3 years, has lost the ability to express functional P and type 1 fimbriae and has, therefore, been able to persist without triggering the host immune response. In contrast to the microorganisms that have acquired genes encoding adhesins for pathogenesis, *E. coli* 83972 is adapted to commensalism through gene loss and mutation [21, 22].

The symptomatic strains of UPEC, which colonize the urinary tract, may ascend towards bladder to cause cystitis, which is usually associated with the classic symptoms of UTIs, that is, pain (painful urination), frequency (frequent urination), and urgency (sudden compelling desire to urinate). However, UTIs can proceed from the bladder, via the ureters to the kidney, to cause pyelonephritis with the possibility of causing irreversible kidney damage and death [23]. Among Gram-negative bacteria, *E. coli* is the most frequent pathogen inducing acute renal failure. Moreover, urological complications, for example after renal transplantation, are associated with UTIs and *E. coli* is the most common clinical isolate [24–26]. Acute allograft injury in the renal transplant population is also associated with both UPEC and clinical diagnosis of upper UTIs [27].

In response to the breach by UPEC into normally sterile urinary tract, the robust host innate response is triggered, including the production of inflammatory cytokines and chemokines. The production of inflammatory mediators results in the rapid recruitment of neutrophils into the bladder lumen and in bacterial clearance [28, 29]. Moreover, the host inflammatory response leads to the exfoliation of infected bladder epithelial cells and generation of reactive nitrogen and oxygen species along with other antimicrobial compounds [30, 31]. These bacteria might also possess multitude of strategies to delay or suppress innate immune response, which facilitate bacterial growth and persistence within the adverse settings of the urinary tract.

This paper summarizes our knowledge about virulence factors of UPEC, the inflammatory responses, and development of tissue damage caused by UTIs.

The mechanisms by which EPEC stimulates proinflammatory response, including the role of Ca^2+^, and influence of this response to development of cystitis and pyelonephritis will also be discussed.

## 2. Virulence Factors of Uropathogenic * E. coli*

UPEC strains encode a number of virulence factors, which enable the bacteria to colonize the urinary tract and persist in face of highly effective host defense. UPEC isolates exhibit a high degree of genetic diversity due to the possession of specialized virulence genes located on mobile genetic elements called pathogenicity islands [4, 32]. Virulence factors of *E. coli* that have been potentially implicated as important to establish UTIs can be divided into two groups: (i) virulence factors associated with the surface of bacterial cell and (ii) virulence factors, which are secreted and exported to the site of action [33].

### 2.1. Surface Virulence Factors

Surface virulence factors of UPEC include a number of different types of adhesive organelles (fimbriae), which promote bacterial attachment to host tissues within the urinary tract.

The presentation of adhesive molecules (adhesins) by UPEC is the most important determinant of pathogenicity. UPEC adhesins can contribute to virulence in different ways: (i) directly triggering host and bacterial cell signaling pathways, (ii) facilitating the delivery of other bacterial products to host tissues, and (iii) promoting bacterial invasion [34].

Type 1 fimbriae are implicated as virulence factors in animal models of urinary tract infection, but their function in human pathology remains unclear [35–39]. Role of the type 1 fimbriae in human disease is difficult to reconcile because they are expressed in both pathogenic and commensal strains [40, 41]. Specifically, there is no significant difference in the *fim *gene frequency between more or less virulent strains in the urinary tract [42]. In the murine UTIs model, the type 1 fimbriae have been shown to enhance bacterial survival, to stimulate mucosal inflammation, and to promote invasion and growth as a biofilm [17, 37, 43–46]. The type 1 fimbriae bind to the urothelial mannosylated glycoproteins uroplakin Ia and IIIa (UPIIIa) via the adhesin subunit FimH, located at the fimbrial tip. This interaction leads to molecular phosphorylation events, which are required for stimulation of signaling pathways involved in invasion and apoptosis and may also contribute to elevation of the intracellular Ca^2+^ level in urothelial cells [4, 17, 47]. Furthermore, Tamm-Horsfall protein (THP) is produced by kidney cells into human urine and can act as a soluble FimH receptor, obstructing bacterial-host cell interaction and limiting the ability of UPEC to colonize the urinary tract [48, 49].

P fimbriae are the second common virulence factor of UPEC, which plays an important role in the pathogenesis of ascending UTIs and pyelonephritis in humans [50–52]. They are responsible for adhesion to mucosal and tissue matrix and for the production of cytokines [53–55]. P fimbriae consist of heteropolymeric fibres composed of different protein subunits, encoded by the *pap*A-K gene operon [56]. These fimbriae recognize kidney glycosphingolipids carrying the Gal *α* (1–4) Gal determinant on renal epithelia via its papG adhesion [2, 57]. Attachment of P fimbriae to this receptor leads to the release of ceramide, which acts as an agonist of Toll-like receptor 4 (TLR4), a receptor involved in activation of the immune cell response [58]. This, in turn, leads to the development of the local inflammation and pain associated with UTIs [59]. Recently, Melican and coworkers [60] have defined previously unknown synergistic functions of the both types of fimbriae, which facilitate bacterial colonization under dynamic *in vivo* conditions. P fimbriae have been shown to enhance early colonization of the tubular epithelium, while the type 1 fimbriae mediate colonization of the center of the tubule via a mechanism that involves inter-bacterial binding and biofilm formation. The heterogeneous bacterial community within the tubule subsequently affects renal filtration leading to total obstruction of the nephron. The obstruction contributes to the full pathophysiology of pyelonephritis [60]. In renal transplant patients, the P fimbriae are also the most common virulence factor and *papG* class II allele is predominant class of the P fimbriae isolated from the patients with acute renal dysfunction. Additionally, in the renal transplant patients with upper UTIs, a majority have an acute allograft injury due to UPEC, which express the P fimbriae [27].

S fimbriae and F1C fimbriae are also implicated in the process of UTIs. Both these types of fimbriae show binding to epithelial and endothelial cell lines derived from the lower human urinary tract and kidney [34, 61, 62]. The S fimbriae may facilitate bacterial dissemination within host tissues and are often associated with *E. coli* strains that cause sepsis, meningitis, and ascending UTIs.

Both the fimbrial Dr and afimbrial Afa adhesins of *E. coli* are associated with UTIs, in particular, with gestational pyelonephritis and recurring cystitis [63–66]. Dr adhesins bind to type IV collagen and decay-accelerating factor (DAF) in the kidney [67]. Dr adhesins have been demonstrated to display a tropism to the basement membrane of the renal interstitium in a mouse model and, therefore, are critical for development of the chronic pyelonephritis [68]. Mutation within the *dra* region encoding for Dr fimbriae prevents development of the tubulointerstitial nephritis.

Adhesins of the Afa family are involved in UTIs [69], and UPEC strains expressing these adhesins have a unique renal tissue tropism [70, 71]. Clinical and experimental findings suggest that *E. coli *strains with Afa adhesins have properties potentially favoring the establishment of chronic and/or recurrent infection [65, 69].

Virulence factors located on the bacterial surface include also the capsule and the lipopolysaccharide (LPS). The capsule is mainly a polysaccharide structure covering and protecting the bacterium from the host immune system [72]. The capsule provides protection against phagocytic engulfment and complement-mediated bactericidal effect in the host. Certain capsular types, for example, K1 and K5, show a molecular mimicry to tissue components, preventing a proper humoral immune response of the infected host [73]. The LPS is an integral component of the cell wall of Gram-negative bacteria. LPS is known to activate host response and to induce nitric oxide and cytokine production [74]. Although LPS of UPEC is important in activation of proinflammatory response in uncomplicated UTIs, it is not clear whether LPS plays a role in mediating a renal failure and acute allograft injury in patients with ascending UTIs. It has been demonstrated in an animal model that the acute renal failure due to LPS depends on the systemic response to LPS and does not depend on expression of functional LPS receptor, TLR4, in the kidney [75]. However, TLR4 is expressed in renal epithelia and in the renal pelvis, and these findings suggest that the ascending infection due to *E. coli *may stimulate the innate immune response associated with the acute allograft injury in patients with UTIs [76, 77].

Flagella, an organelle responsible for bacterial motility, is involved in the interaction of various pathogenic *E. coli *strains with epithelial cells. Flagellated UPEC cause 70–90% of all urinary tract infections, and their pathogenesis involves contact between the bacteria and epithelial cell surface of the urinary tract. The pyelonephritis-associated *E. coli *strains may invade renal collecting duct (CD) cells through flagellin, and the flagellin acts as an invasin in this process [78]. Other studies have suggested that *E. coli* flagella may be of importance in allowing the bacteria to ascend from the bladder and to initiate kidney infection in humans. The use of an antibody against the flagella could prevent the spread of UPEC into the kidneys [79].

### 2.2. Secreted Virulence Factors

Toxins are important virulence factors in a variety of *E. coli*-mediated diseases. Production of toxins by colonizing *E. coli* may cause an inflammatory response, a possible pathway for UTIs symptoms. The most important secreted virulence factor of uropathogenic *E. coli* is a lipoprotein called *α*-haemolysin (HlyA), which is associated with upper UTIs such as pyelonephritis [73]. The HlyA is a pore-forming toxin, which belongs to the family of RTX (repeats in toxin) toxins that are widespread among the Gram-negative pathogens [80, 81]. This toxin has been shown to exert dual concentration-dependent activities on primary epithelial cells originating from renal proximal tubules [82]. At high concentrations, HlyA is able to lyse erythrocytes and nucleated host cells, a process that may enable extraintestinal pathogens like UPEC to better cross mucosal barriers, damage effector immune cells, and gain enhanced access to host nutrients and iron stores [73, 83, 84]. At low concentrations, HlyA can induce the apoptosis of target host cells, including neutrophils, T lymphocytes, and renal cells, and promote the exfoliation of bladder epithelial cells [85–88]. HlyA has also been shown to induce Ca^2+^ oscillations in renal epithelial cells, resulting in increased production of IL-6 and IL-8 [89]. Approximately 50% of all cases of pyelonephritis, which leads to renal complications, are caused by HlyA. The *E. coli*-expressed *α*-hemolysin may induce endothelial damage and renal vasoconstriction, for example, by the intrarenal release of endothelin [90]. Moreover, permanent renal scarring is a common complication following HlyA *E. coli* infection [91, 92] and may be independent of bacterial adherence properties [93]. Moreover, it has been shown that HlyA and other *E. coli* toxins cause inducible nitric-oxide-synthase-(iNOS-) mediated cell membrane injury and apoptosis, a process that is regulated by extracellular signal regulated kinase (ERK) independently of the p53 pathway [94].

The cytotoxic necrotising factor 1 (CNF1) [95, 96] is produced by one-third of all pyelonephritis strains and may also be involved in kidney invasion. *In vitro*, this protein is secreted by *E. coli *and stimulates actin stress fibers formation and membrane ruffle formation in a Rho GTPase-dependent manner, resulting in the entry of *E. coli *into the cells. However, the detailed role of CNF1 in invasion processes during pyelonephritis remains unclear and is a matter of debate [30]. *In vitro* studies have also showed that CNF1 interferes with polymorphonuclear phagocytosis and evokes apoptotic death of bladder epithelial cells [97, 98]. *In vivo*, CNF1 may lead to bladder cell exfoliation and to enhanced bacterial access to underlying tissue [97].

Secreted autotransporter toxin (SAT) is a virulence factor of pyelonephritis *E. coli* strains. SAT has a toxic activity against cell lines of bladder or kidney origin and, thus, may be important for pathogenesis of UTIs [99, 100]. Moreover, the cytolethal distending toxin (CDT) could be also considered as a virulence factor in UTIs caused by *E. coli* [101, 102]. Toll/interleukin (IL-1) receptor (TIR) domain-containing protein (Tcp) represents a novel class of virulence factors, which are able to subvert TLR signaling to gain a survival advantage during UTIs. Importantly, UPEC encoding Tcp promotes bacterial survival and kidney pathology *in vivo *[103].

## 3. Host Defenses against UPEC Colonization of the Urinary Tract

The urinary tract is a typically sterile environment, which is maintained by a variety of host mechanisms to prevent bacterial colonization and survival. Most of the pathogenic bacteria, which cause UTIs, are from the host own bowel flora and enter the bladder via the urethra. Uroepithelial adherence is critical for establishment of UTIs. UPEC strains possess an impressive repertoire of adhesins that enable the bacteria to aggregate and adhere to the cellular surfaces [34, 57, 62, 69, 104]. Consequently, the first line of host defense against UTIs is concentrated on preventing UPEC adherence to the bladder mucosa.

### 3.1. Primary Bladder Defenses

The urinary tract has a number of specialized defenses against bacterial colonization, keeping the urine sterile. The bulk flow of the urine through the bladder and micturition can work to rinse away nonattached or weakly adherent microbes from bladder surface [105]. Secretion of glucosamines by bladder transitional cells prevents bacterial adherence by forming a mucin layer. The low pH and presence of salts, urea, and organic acids in urine can reduce bacterial survival within the urinary tract. The Tamm-Horsfall glycoprotein acts as an antiadhesive urinary factor by binding to UPEC expressing type 1 fimbriae and forming the complex, which is then cleared by voiding [106–109]. Defensins (a group of small, highly cationic antimicrobial peptides) are also produced in the urinary tract after exposure to the pathogens. Defensins have the capacity to kill bacteria, fungi, and some encapsulated viruses. These peptides attach to the anionic phospholipids on the cell wall of pathogens and disrupt their cell membrane function, increasing cell permeability and causing cell death [110].

### 3.2. Host Response and Consequences of UPEC Adherence and Invasion

If a microbe manages to sidestep these constitutive host defenses and makes contact with the urothelium, its continued presence can trigger the activation of additional host defense mechanisms, leading to exfoliation of infected bladder epithelial cells and inflammation.

#### 3.2.1. Exfoliation of Infected Cells

A key feature of inflammation during UTIs is a disruption of the urothelial integrity due to the exfoliation and subsequent excretion of infected eukaryotic cells [111, 112]. This FimH-dependent exfoliation process occurs via an apoptosis-like pathway that involves the activation of caspases, cysteine proteases implicated in the execution of apoptosis, and DNA fragmentation [16, 113]. The importance of this response as a host defense is illustrated by the fact that the inhibition of exfoliation with a pan-caspase inhibitor dramatically reduces bacterial secretion from the bladder early in the infection. The bacteria, which manage to escape from dying superficial cells before the exfoliation process is completed, can go on to infect surrounding and underlying tissue [114]. This may not only promote bacterial dissemination within the urinary tract but could also allow UPEC to enter a sheltered environment within the bladder, where bacteria can persist for long time. Thus, exfoliation is a powerful mechanism of eradication of both attached and internalized bacteria from the bladder epithelium.

#### 3.2.2. Inflammation

Upon successful adherence to the uroepithelium, the presence of bacteria and bacteria factors/products within the urinary tract can trigger rapid and robust responses from the host.

Infection with UPEC elicits both innate and adaptive immune responses, although an efficient host defense against urinary tract infection is reliant upon an early activation of the innate immune response [115, 116]. This response is characterized by the production of a number of proinflammatory mediators, including cytokines and chemokines [117–119]. Bladder and kidney epithelial cells appear to be a major source of interleukin-6 (IL-6) and interleukin-8 (IL-8) after infection with UPEC, which are important in the development of local tissue damage [119–123]. IL-6 possesses a variety of proinflammatory functions, including activation of signals involved in neutrophils recruitment and production of acute phase proteins [124]. A high urinary concentration of IL-6 during acute phase of pyelonephritis has been shown to correlate with an increased risk of permanent renal scars [125]. IL-6 gene polymorphisms have been connected to susceptibility to UTIs, but not scar formation in children [126]. IL-8 is a potent neutrophil chemotactic molecule. In humans, the induction of IL-8 after infection with UPEC correlates with appearance of neutrophils in the urine [119]. Neutrophil recruitment to the site of infection has been shown to be critical for bacterial clearance from both the bladder and kidney, and the presence of neutrophils in the urine is a hallmark of UTIs. However, their action may also lead to the local tissue damage [116]. Interactions between the neutrophil receptor CD11/CD18 and the adhesion molecule ICAM-1 on bladder epithelial cells seem to be critical for neutrophil migration into the urothelium [119].

Recently, it has been shown that IL-17A, an immunomodulatory cytokine, is involved in the innate immune response to UTIs. A key source of IL-17A production appears to be *γδ*-positive cells. IL-17A importance for the innate immune response has been demonstrated by a defect in acute clearance of UPEC in IL-17A^−/−^ mice. This clearance defect is likely a result of deficient cytokine and chemokine transcription and of impaired macrophage and neutrophil influx during infection [127].

In conclusion, explanation of the signaling pathways involved in the neutrophil recruitment into the bladder and kidneys highlights the importance of cytokines and chemokines.

Importantly, activation of the innate immune response has a dual effect: it is necessary for eradication of pathogenic bacteria but may also lead to tissue damage and permanent scarring.

**(1) Host Signaling in Response to UPEC Recognition**

**Figure 1:**
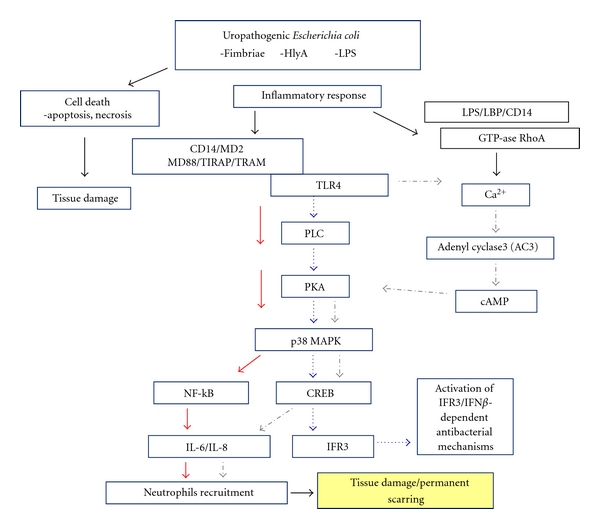
Model of UPEC-induced signaling cascades in urothelial cells. Bacterial adherence to uroepithelium results in an epithelial inflammatory response including local production of chemokines and cytokines or cell death via apoptosis. Activation of TLR4 through virulence factors, including LPS and fimbriae, triggers the response, which involves activation of kinases and subsequent translocation of different transcriptional factors such as NF-*κ*B, CREB, and IFR3 into the nucleus. HlyA (through LPS-LBP-CD14 complex) is delivered to the cell membrane and activates there GTPase RhoA, which is required for Ca^2+^ signaling. Specific HlyA-induced Ca^2+^ oscillations lead to activation of NF-*κ*B and synthesis of IL6/IL-8. Chemokine production leads to recruitment of neutrophils that kill the bacteria by producing the cytotoxic substances. Tissue damage, cell death, and permanent scarring result in excessive inflammatory response.

The activation of the innate immune response in the urinary tract is dependent on recognition of bacterial components/products by TLRs [128–131]. In recent years, it has become clear that the immune activation of bladder and kidney epithelial cells depends on TLRs, including TLR4, TLR5, and TLR11 [74, 77, 128, 129, 132]. Recognition of virulence factors by TLRs stimulates signaling pathways resulting in activation and translocation of NF-*κ*B. In the nucleus, NF-*κ*B activates the transcription of proinflammatory genes, such as those encoding IL-6 and IL-8. NF-*κ*B is one of the major transcription factors required for induction of proinflammatory response. However, in response to bacterial products, the bladder epithelial cells can activate NF-*κ*B-independent signaling pathway (see below) [133]. More recently, the involvement of interferon regulatory transcriptional factor IRF3 in antibacterial defense and immunoregulation by TLRs has received more attention [134–137]. Possession of multiple signaling pathways for production of cytokines is advantageous in case when bacteria have an ability to suppress certain signaling events important for cytokine and chemokine production (Figure 1).

TLR4 attracts most of the attention in context of the mechanisms of immune defense in the urinary tract. TLR4 is responsive to LPS of Gram-negative bacteria. TLR4 is expressed on the epithelial cells throughout the urinary tract and is required to mount an effective inflammatory response after infection with UPEC [75]. However, both the role of UPEC LPS and an existence of another bacterial trigger of TLR4 signaling are an area of debate [54, 74, 117]. Several reports have claimed that uroepithelial cells are refractory to LPS stimulation and have argued that, instead, bacterial fimbriae such as the type 1 fimbriae, P fimbriae drive the induction of cytokines in these cells [130, 138]. Studies using the human kidney cell line indicate that TLR4-mediated signaling pathway in response to UPEC is dependent on P fimbriae and can be initiated independently of LPS/CD14 [54, 58, 130, 139]. Recently, the new IRF3-dependent signaling pathway, which leads to induction of the innate immune response in TLR4- and P-fimbriae-dependent manner, has been described. Mechanistic details regarding this phenomenon include P fimbriae binding to surface glycosphingolipids (GSLs) and subsequent release of the GSL membrane-anchoring domain, ceramide [58, 140]. This molecule appears to act as a TLR4 agonist and the putative intermediate for TLR4 signaling initiated by P fimbriae [58]. Ceramide-induced TLR4 signaling causes rapid phosphorylation of proteins, including TRAM, PLC, Fyn, PKA, p38 MAP kinase, ERK1/2, and CREB, which are implicated in nuclear translocation of IFR3 and activation of IRF3/IFN*β*-dependent antibacterial effector mechanisms [141]. The IRF3-dependent signaling pathway is essential for the host defense and is critical for distinguishing pathogens from normal flora at the mucosal barrier. In the absence of IRF3 (in the *Irf3*-knockout mice) the acute mortality, bacterial burden, abscess formation, and renal damage have been observed, consistent with the need for this pathway to maintain a functional antimicrobial defense. Relevance of IRF3 pathway for human disease was supported by data concerning polymorphic IRF3 promoter sequences, which differ between children with severe, symptomatic kidney infection and children who were asymptomatic bacterial carriers [141]. Recently, the FimH tip adhesin of type 1 fimbriae has been shown to interact directly with TLR4—an additional means for LPS-independent stimulation of TLR4 [142, 143].

However, other results underline the important role for LPS and TLR4 in the stimulation of bladder epithelial cells by type 1 fimbriae *E. coli *[74, 118]. Stimulation of TLR4 by LPS and type 1 fimbriae correlates with the level of CD14 expression on bladder cells [117, 133, 144]. Interestingly, CD14 expression is localized to the submucosa, and this may suggest that uroepithelial cells exposed to the lumen have little to no CD14 expression and, therefore, cannot respond efficiently to LPS alone. These results support a role for both LPS-dependent and cooperative TLR4 stimulation by UPEC fimbriae. The inflammatory response to LPS is mediated via specific LPS-binding protein, accessory molecules CD14 and MD2, and TIR domain. The TIR domain of TLR4 interacts with the adaptors MyD88 and TIR domain containing adaptor protein (TIRAP) [145]. Activation of TLR4 is known to lead finally to p38 MAPK activation and nuclear translocation of NF-*κ*B, and transcription of inflammatory response genes (Figure 1).

TLR5 plays a crucial role in host defense to UPEC infection by mediating flagellin-induced inflammatory responses in the bladder [128, 146]. Hence, a study in TLR5^−/−^ mice challenged with UPEC demonstrated a decreased inflammatory response early after urethral bacterial infection and, subsequently, a concomitantly increased bacterial burden in both the bladder and kidney in these mice [128].

Recently, a new TLR11 expressed in the kidney, bladder, and liver of mice was discovered [129]. Mice lacking TLR11 are highly susceptible to infection of the kidneys by UPEC indicating a potentially important role for TLR11 in preventing infection of internal organs of the urogenital system [129]. In humans TLR11 might not play a significant role due to the abundance of stop codons occurring in the human TLR11 gene.

**(2) Genetic Polymorphism**

Gene polymorphisms have been shown to have an influence on the inflammatory response of uroepithelium to bacteria as well as on susceptibility to kidney damage. Polymorphism of the TLR4 gene is associated with hyporesponsiveness to LPS, absence of neutrophil recruitment, and delayed clearance of bacteria from the urinary tract in mice [116]. Polymorphisms in the TLR4 gene may also have a role in the inflammatory response in humans. Lorenz et al. [147] have suggested that the TLR4 Asp299Gly allele predisposes individuals to septic shock and a higher prevalence of Gram-negative bacteremia. Recently, Ragnarsdóttir et al. [148] have described a new concept for human TLR variation, based on TLR4 promoter polymorphisms that influence gene expression dynamics *in vitro* and the innate immune response dynamics in patients with asymptomatic bacteriuria. They suggest that reduced TLR4 expression attenuates the innate mucosal response, thus promoting an asymptomatic carrier state rather than severe disease [148]. Hawn et al. [149] have shown that TRL5 stop codon polymorphism abolishes flagellin signaling and is associated with increased susceptibility to Legionnaire's disease. These murine studies support a hypothesis that individuals, who possess this TLR5 variant, will also be more susceptible to UTI. Polymorphisms in cytokine genes have also been connected to frequency and severity of urinary tract infections, probably as a result of variability in immune responses in such patients [150, 151]. For example, the gene polymorphism associated with TNF*α* has been observed in patients with reflux nephropathy [150]. Similarly, Cotton et al. [151] have shown that variability in the transforming growth factor-*β*1 (TGF-*β*1) gene predisposes individuals to postinfectious renal scarring. As previously described, TGF-*β*1, the production of which is activated by other cytokines (IL-6, IL-8, IL-1, and TNF-*α*), leads to the extracellular matrix deposition through an increased synthesis of matrix proteins, and at the same time by decreasing matrix protein degradation following a suppression of protease expression and an increased production of tissue inhibitor of matrix metalloproteinases (TIMPs). All of these facts support the hypothesis that TGF-*β*1 is an important factor in the pathogenesis of renal parenchymal scarring following UTI. Variable constitutive or induced expression of TGF-*β*1 protein could occur as a consequence of variability in the TGF-*β*1 gene, which in turn could be associated with differential effects on cellular growth or extracellular matrix deposition leading to renal parenchymal scarring [152].

#### 3.2.3. Ca^2+^Signaling during UPEC Infection is Important for Innate Immune Response and Pathogenesis of Infection

Several lines of evidence indicate that Ca^2+^-dependent signaling pathway interacts with signal transduction pathways implicated to the innate immune response. It supposes that any effect on Ca^2+^ regulation is likely to have some influence on the innate immune response [153]. Interestingly, expression of IL-8 has been shown to be under control of artificially induced Ca^2+^ oscillations due to frequency-modulated expression of the transcription factor NF-*κ* B. Moreover, IL-6 is responsible for activation of response that involves not only NF-*κ* B pathway, but also other signaling, which implicates two secondary messengers, Ca^2+^ and cAMP, and mobilizes a transcriptional element known as cAMP response element-binding protein (CREB) [133]. Different bacterial virulence factors can induce an increase of Ca^2+^ concentrations in the host cells [154–156]. *α*-Haemolysin of UPEC elicits oscillatory fluctuations of intracellular Ca^2+^ when present in the sublytic concentrations [89, 157]. It has been shown that Ca^2+^ oscillations, corresponding to a periodicity of 12 min, specifically stimulate production of IL-6 and IL-8 in human renal epithelial cell [89].

Experiments designed to identify components of the intracellular signaling pathway leading to Ca^2+^ oscillations revealed that specific activity of HlyA is required together with an activation of L-type Ca^2+^ channels to obtain Ca^2+^ oscillations. IP3-receptor-gated Ca^2+^ stores of the endoplasmic reticulum are involved [157, 158]. 

HlyA has long been known to physically interact with LPS, which plays an indirect role in the cytolytic activity of HlyA in erythrocytes and in epithelial cells [159–161]. Moreover, the ability of Hly to induce Ca^2+^ oscillations has been suggested to require LPS as a cofactor. Månsson et al. [161] have shown that the LPS and HlyA complex exploits the CD14/LPS-binding protein (LBP) recognition system to bring HlyA to the cell membrane, where intracellular Ca^2+^ signaling is initiated via specific activation of the small GTPase RhoA. Additionally, HlyA-induced Ca^2+^ signaling has been found to occur independently of the LPS receptor TLR4. Also, the cytolytic effect triggered by exposure of cell to high HlyA concentrations occurs independently of the CD14/LPS-LBP complex, suggesting that cytolysis is induced through mechanism different from that used for induction of Ca^2+^ oscillations [161].

**(1) TLR4-Mediated NF-B-Independent Signaling**

Studies on TLR4 signaling have revealed the existence of a distinct TLR4-mediated signaling pathway leading to IL-6 secretion. This pathway is present in the bladder epithelial cells and is activated upon exposure to LPS [133]. Interestingly, this signaling is independent of the pathway involving the NF-*κ*B and contains two well-known secondary messengers, Ca^2+^ and cAMP, which mobilize transcription factor CREB. The CREB binds to cAMP-response element (CRE) promoter sites to regulate the transcription of numerous genes in response to a diverse stimuli [133, 162]. Intracellular cAMP is an important second messenger in several signaling pathways, including IL-6 response. The increase in cAMP following bacterial exposure depends on both bacterial-associated LPS and increase of intracellular Ca^2+^. This *E. coli*-induced Ca^2+^-dependent cAMP production strictly correlates with activation of adenyl cyclases (ACs). Because there are currently ten known isoforms of mammalian ACs, it is noteworthy that of the four ACs isoforms expressed in bladder epithelial cells only adenyl cyclase 3 (AC3) is known to be activated by increase in intracellular Ca^2+^ [163, 164]. *E. coli*-induced Ca^2+^ spike leads to AC3-mediated increase in cAMP, protein kinase A (PKA) activation, and phosphorylation of the CREB [162]. Upon phosphorylation, CREB promotes transcription of a number of genes, including IL-6 and IL-8 (Figure 1) [162, 165, 166]. Using selective blockade of different signaling pathways, it has been determined that the activation of cytokine secretion by UPEC *E. coli* might even be faster via the CREB than via the NF-*κ*B pathway [133]. The capacity of the bladder epithelial cells to mobilize the secondary messengers and to evoke the rapid IL-6 response could be critical in their role as the first responders to microbial challenge in the urinary tract.

**(2) UPEC FimH-Induced Elevation in Urothelial Ca**

Activation of the host signal transduction cascades by bacterial attachment is a well-recognized consequence of the host-pathogen interactions [167], and urothelial signaling events associate with UPEC invasion and urothelial cell apoptosis [168]. FimH is involved in adhesion, invasion, and apoptosis of urothelial cells and initiates bladder pathology by binding to the uroplakin receptor complex. Recently, hitherto undiscovered signaling role for the UPIIIa in bacterial invasion and apoptosis has been presented. The UPIIIa is the only major uroplakin with a potential cytoplasmic signaling domain. In response to FimH adhesin binding, the UPIIIa cytoplasmic tail undergoes phosphorylation on a specific threonine residue by casein kinase II, followed by an increase in intracellular Ca^2+^ concentration [47]. FimH-mediated Ca^2+^ elevation occurs as a result of Ca^2+^ release from intracellular stores and by influx from extracellular sources. Ca^2+^ elevation promotes global responses critical to UPEC pathogenesis, including cytokine stimulation, membrane trafficking, and apoptosis [133, 156].UPEC-induced UPIIIa signaling is a critical mediator of the pathogenic cascade induced in the host cell and is a novel therapeutic target.

#### 3.2.4. UPEC Escapes from Host Innate Immune Response

Several lines of evidence suggest that UPEC might possess strategies to delay, attenuate, suppress, or subvert the activity of components of the innate immune response in the urinary tract, especially early in infection [16, 113, 169–172]. Suppression of NF-*κ*B activation by UPEC results in enhanced type-1-fimbriae-mediated apoptosis of urothelial cells and in decreased levels of inflammatory cytokines production as well as neutrophil recruitment [113, 170, 172]. By hindering host cytokine expression and ensuing inflammatory responses, UPEC may be better able to establish itself and multiply within the cells and tissues of the urinary tract.

UPEC infection in the murine bladder upregulates expression of the suppressor of cytokine signaling 3 gene [112]. This phenomenon might represent a conserved strategy to subvert host defenses, allowing UPEC to survive in the bladder. Additionally, several genes involved in LPS biosynthesis (e.g., *rfa* and *rfb*) and *surA *gene implicated in biogenesis of outer membrane proteins are important for the phenotype, suggesting that alternation in LPS structure may underline the notstimulatory properties of UPEC [169, 172]. Hilbert et al. [173] have shown that the bladder epithelial cells secrete IL-6 and IL-8 in response to nonpathogenic *E. coli *but are unable to mount the same cytokine response following exposure to UPEC, revealing dominant suppression of the innate immune response through a pathway partially independent of LPS and TLR4.

Recently it has been shown that TIR homologous protein TcpC inhibits MyD88-dependent gene expression in infected CF073 human uroepithelial cells [103, 174]. The effects of TcpC on bacterial persistence were attenuated in Trif^−/−^ or IL-1^−/−^ mice, and innate immune responses were increased, confirming that Trif and IL-1-dependent targets might be involved *in vivo*, in addition to MyD88. Loss of TcpC led to decreased bacterial burden in kidneys and to reduced renal damage, showing the importance of these proteins in pathogenesis of the urinary tract infection.

Additionally, it has been shown that UPEC downregulates neutrophil activity, a phenotype, which is important during initiation and progression of infection, or for subsequent establishment of UPEC reservoir in the bladder [175].

The ability of UPEC to suppress the innate immune response plays a role in persistence of pathogens within urinary tract. However, identifying the gene(s) and factors that are involved in this process will contribute to understanding of UPEC pathogenesis and provide potential novel diagnostic and therapeutic targets.

#### 3.2.5. Complications Associated with UTI

Renal bacterial infections are common infectious diseases that can impair renal function and/or lead to the renal tubulointerstitial nephritis. Bacteria can invade the kidneys via the systemic circulation or by local retrograde infection. They can cause severe renal dysfunction and are associated with various kidney diseases, such as IgA nephropathy, renal vasculitis, and lupus nephropathy in postinfectious glomerulonephritis [176–178].

 In the previous sections we have described mechanisms involved in induction of proinflammatory response to UPEC. The innate immune system recognizes the virulence determinants of pathogens via receptors and activates the line of defense against pathogens. However, if functionality of the proinflammatory response is delayed or suppressed, bacteria after colonization the bladder can ascend the ureters and the kidneys. At this juncture, a risk of permanent renal scarring exists, and bacteria can access the bloodstream [179]. In response to UPEC, the renal cells activate proinflammatory mediators, which play essential roles in the first line of defense against pathogens in the kidney. However, when this response is excessive, acute, or chronic pyelonephritis may occur, leading to severe damage and renal failure [180–182]. The acute pyelonephritis is an acute inflammation of the renal parenchyma and pelvis associated with bacterial infection. Clinically, the acute pyelonephritis is a severe form of urinary tract infection with symptoms that range from mild discomfort to life-threatening illness or death [183]. Complications may result in chronic renal scarring (atrophic pyelonephritis or reflux nephropathy) and impairment of renal function [184, 185].

The chronic pyelonephritis has been defined as a destructive inflammatory process involving both the pyelocaliceal system and renal parenchyma [186]. The renal parenchymal lesions include tubular atrophy, interstitial inflammation, and interstitial fibrosis. Parenchymal lesions may be relentlessly progressive and may result in end-stage kidney. UTIs caused by UPEC are also the most frequent infectious complications in renal transplant patients and can impair long-term renal graft function [187].

In conclusion, further studies are required to determine the mechanisms by which virulence factors of UPEC interact with the kidneys and lead to the renal failure as well as to the deterioration of renal allograft function. Concurrently, better understanding of functions of virulence factors implicated in renal damage could open the way to control the immune response in the kidney and may be helpful for the development of effective therapies for *E. coli*-caused kidney diseases.

## 4. Conclusion

Among the Gram-negative bacteria, UPEC is the pathogen most frequently associated with UTIs. UPEC, which colonizes the urinary tract, may ascend towards bladder to cause cystitis. Left untreated, bacteria ascend the ureters to the kidney and establish a secondary infection, acute pyelonephritis with the possibility of causing irreversible kidney damage leading to kidney failure and death.

 The specific host-pathogen interactions are required to activate inflammation based on production of cytokines and chemokines by epithelial cells of the urinary tract. Expression of adhesive organelles allows UPEC to bind and to invade the host cells and tissues within the urinary tract. Moreover, deployment of an array of toxins provides UPEC with the means to inflict an extensive tissue damage, facilitating bacterial dissemination as well as releasing host nutrients and disabling the immune effector cells. Recognition of bacterial products by TLRs activates NF-*κ*B-dependent signaling pathway, leading to translocation of NF-*κ*B into the nucleus and to expression of proinflammatory mediators, such as IL-6 and IL-8. Additionally, the bladder epithelial cells activate other NF-*κ*B-independent signaling pathways, which results in Ca^2+^, cAMP, PKA, and CREB activation. UPEC can modulate Ca^2+^ signaling in the urothelial cells through several mediators. Altered urothelial Ca^2+^ signaling can modulate gene transcription, can stimulate cytokine expression in response to LPS and TLR4, and can be initiated by the interaction of UPEC adhesin FimH with the integral membrane protein UPIIIa to cause urothelial invasion and apoptosis in the bladder. UPEC possesses also an ability to interrupt the proinflammatory NF-*κ*B signaling. These findings suggest more complicated sequence of early events in the pathogenesis of UTIs that may enhance the potential for recurrent UTIs. Moreover, recently described new IRF3 signaling indicates that the genetic variation in IRF3 influences individual susceptibility to the kidney infection and might serve as a new tool for future risk assessment in this patient group.

## Abbreviations

Acs: Adenyl cyclases
CD14: Cluster of differentiation 14
CDT: Cytolethal distending toxin
CNF1: Cytotoxic necrotising factor 1
CREB: cAMP-response-element-binding protein
DAF: Decay-accelerating factor
ERK: Extracellular signal regulated kinase
GSL: Glycosphingolipid
IRAKs: Interleukin 1 receptor-associated kinases
LBP: Lipopolisaccharide-binding protein
LPS: Lipopolisaccharide
MCP-1: Monocyte chemotactic protein-1
MD2: Myeloid differentiation factor 2
MIP2: Macrophage inflammatory protein 2
MyD88: Myeloid differentiation factor-88
NF-*κ*B: Nuclear factor kappa B
PKA: Protein kinase A
SAT: Secreted autotransporter toxin
TIR: Toll-interleukin 1 receptor
Tcp: TIR domain-containing protein
TIRAP: TIR domain-containing adaptor protein
THP: Tamm-Horsfall protein,
TLRs: Toll-like receptors
TRIF: TIR domain-containing adaptor protein inducing IFN*β*
TRAM: TRIF-related adaptor molecule.
